# Predictive Markers of Bronchial Hyperreactivity in a Large Cohort of Young Adults With Cough Variant Asthma

**DOI:** 10.3389/fphar.2021.630334

**Published:** 2021-04-19

**Authors:** Mario Malerba, Beatrice Ragnoli, Danila Azzolina, Paolo Montuschi, Alessandro Radaeli

**Affiliations:** ^1^ Department of Translational Medicine, University of Piemonte Orientale, Novara, Italy; ^2^ Respiratory Unit, S. Andrea Hospital, Vercelli, Italy; ^3^ Pharmacology, Faculty of Medicine, Catholic University of the Sacred Heart, Roma, Italy; ^4^ ASST Spedali Civili di Brescia, Department of Emergency, Brescia, Italy

**Keywords:** bronchial hyperreactivity, fractional exhaled nitric oxide, forced expiratory flow, sputum eosinophils, cough variant asthma, bronchial provocation tests

## Abstract

Cough variant asthma (CVA), a common asthma phenotype characterized by nonproductive cough and bronchial hyperreactivity (BHR), is usually detected by bronchial provocation tests (BPTs) which are time**-**consuming, expensive, and unsafe. The primary study objective was to provide proof of concept for the use of fractional exhaled nitric oxide (F_E_NO), eosinophil count percentage in induced sputum (sEOS%), forced expiratory flow between 25 and 75% of forced vital capacity (FEF_25–75%_) % predicted value, and FEF_25–75%_ z-scores as surrogate markers predicting BHR in young adults with suspected CVA; the secondary objective was to compare the diagnostic performance of the various techniques. Three hundred and ten subjects (median age 24 years) were included in a cross-sectional study. Subjects were characterized as BHR positive (POS) (*n* = 147) or BHR negative (NEG) (n = 163) according to methacholine BPT. Classification accuracies were expressed as areas under the receiver operator characteristic curves (AUC). Compared with BHR NEG, FEF_25–75%_ % predicted value and FEF_25–75%_ z-scores were lower in the BHR POS group (*p* < 0.001), whereas F_E_NO (*p* < 0.001) and sEOS% were higher (*p* < 0.001). AUC values for detecting BHR were as follows: F_E_NO, 0.98 (SD = 0.02); sEOS%, 0.98 (SD = 0.02); FEF_25–75%_ % pred, 0.93 (SD = 0.05); FEF_25–75%_ z scores, 0.92 (SD = 0.05). Optimal cutoff values (OCV) for BHR prediction were as follows: F_E_NO, 32.7 ppb (sensitivity = 0.93, specificity = 0.96), sEOS%, 3.80% (sensitivity = 0.94, specificity = 0.94), FEF_25–75%_ % predicted value, 80.0% (sensitivity = 0.90, specificity = 0.87), and FEF_25–75%_ z-score, −0.87 (sensitivity = 0.89, specificity = 0.87). Non-invasive/semi-invasive airway inflammatory or small airway functional measures might be used as surrogate markers predicting BHR in young adults with suspected CVA.

## Introduction

Asthma is characterized by chronic airway inflammation, bronchial hyperreactivity (BHR), and episodes of bronchoconstriction clinically presenting as variable and recurring cough, dyspnea, and wheezing (ginasthma, 2020). Asthma is a heterogeneous disease, including a broad spectrum of diseases described as various phenotypes (Haldar et al., 2008). Cough variant asthma (CVA), a frequent asthma phenotype, is characterized by a cough as a prevalent symptom and BHR (Corrao et al., 1979). The presence of BHR is generally detected with bronchial provocation tests (BPTs), a positive response to bronchodilators or both (Irwin et al., 2006; Achilleos, 2016). BPTs are the gold standard, but expensive, time consuming, and unsafe as they are potentially able to induce severe bronchospasm (Coates et al., 2017). Simpler, safer, and more rapid predictive methods would be relevant to clinical practice (Bao et al., 2018) as they would facilitate the identification of those patients with suspected CVA who need to be referred for BHT.

Fractional exhaled nitric oxide (F_E_NO) is a non-invasive, standardized, safe, simple, and well-accepted surrogate marker of airway inflammation (Jatakanon et al., 1998b; ElHalawani et al., 2003; Berkman et al., 2005; Malerba et al., 2008). F_E_NO is elevated in subjects with atopic asthma (Ricciardolo et al., 2004) and correlates with sputum eosinophilia before and after glucocorticoid treatment (Jatakanon et al., 1998b; Malerba et al., 2008), and with bronchoalveolar lavage (BAL) eosinophil counts (Lim et al., 1999); a strong correlation between F_E_NO concentrations and BHR has been observed in children with asthma (Ciprandi et al., 2010). F_E_NO levels were also found correlated with BHR in apprentices exposed to occupational risk for asthma (Tossa et al., 2010).

Increasing evidence shows that inflammation of small airways (<2 mm diameter) and surrounding alveolar tissue and small airway function play a pivotal pathophysiological role in cough exacerbation, nocturnal attacks, and exercise-induced wheeze (Van Der Wiel et al., 2013). Forced expiratory flow between 25 and 75% of forced vital capacity (FEF_25–75%_) has been found to correlate with functional imaging assessment of small airway function (Jain et al., 2005) and proposed as an early marker for peripheral airway airflow limitation (<2 mm) (McFadden and Linden, 1972; Terra Filho et al., 1986; Perez et al., 2013) and eosinophilic inflammation (Malerba et al., 2016). Using computed tomography airway morphometric analysis, (Niimi et al., 2000), several studies have shown a good correlation between FEF_25–75%_ and the High-Resolution computed tomography finding of air trapping. Recently, FEF_25–75%_ has been shown to be feasible parameter for identifying small airway dysfunction early in CVA patients (Yuan et al., 2019). Moreover, in a cross sectional study, reduced FEF_25–75%_ was associated with increased frequency of respiratory symptoms, greater healthcare utilization and higher levels of biomarkers of distal airway inflammation, including F_E_NO and sputum eosinophils (Riley et al., 2015).

Eosinophil differential count in induced sputum (sEOS%), a semi-invasive technique, is a standardized, recommended, evidence-based, direct measure of airway inflammation and its use is reported into the most relevant guidelines (Pin et al., 1992). sEOS% counts increase during asthma exacerbations (Pizzichini et al., 1999) and, similarly to F_E_NO concentrations, decrease after treatment with corticosteroids (Jatakanon et al., 1998a). The primary objective of this study was to provide proof of concept for the use of various noninvasive/semi-invasive inflammatory or functional measures, including F_E_NO, sEOS% and FEF_25–75%_, as surrogate markers predicting BHR in a cohort of young adults with suspected CVA and maintained lung function as reflected by normal forced expiratory volume in 1 s percentage of predicted (FEV_1_%) values; secondary study objective was to compare their diagnostic performance. We chose to study subjects aged from 18 to 45 years to minimize the impact of confounding factors related to airways aging and possible co-morbidities associated with older ages on study outcomes.

## Methods

### Subjects and Study Design

We performed a cross-sectional study of data collected from 310 adult subjects aged from 18 to 45 years referred for cough to the Respiratory Medicine Unit of the Department of Internal Medicine, University of Brescia and to the Department of Translational Medicine, University of Piemonte Orientale, Respiratory Unit of Vercelli’s Hospital, Italy, in an out-subject setting from January 2016 to March 2018.

Inclusion criteria were as follow: suspected CVA with cough as a predominant symptom, chest tightness, dyspnea or wheezing with nocturnal awakenings for >3 weeks; normal chest X-ray; maintained lung function as reflected by FEV_1_% > 80% of predicted values with spirometric measurement. Subjects were excluded if they met the following criteria: upper respiratory infection during the previous 6 weeks, use of systemic and/or inhaled corticosteroids during the previous 6 weeks, current or past history of smoking, any significant medical condition, a prior asthma diagnosis and the usual contraindications to methacholine challenge tests. No subject was under antihistamines and no subject had symptoms of allergic rhinitis at the time of the inclusion. The study was approved by the Local Center Ethics Committees (N 0770-2016 and N 035 -2017) and all the subjects gave their written informed consent. Recruited subjects underwent the following procedures: clinical examination; symptom evaluation; skin prick testing; pulmonary function tests; methacholine challenge test; F_E_NO measurement; sputum induction, and sEOS% count. Interventions were performed in the following order to reduce the effect of bronchoconstriction on FENO: FENO measurement, spirometry, methacholine challenge and sputum induction (American Thoracic Society, European Respiratory Society, 2005).

This study was conducted in agreement with the STROBE statement for observational studies (von Elm et al., 2008).

### Skin Prick Test

Allergy was assessed by skin prick test positivity to the most common respiratory allergens as stated by the European Academy of Allergy and Clinical Immunology (Anonymous, 1989).

### Pulmonary Function Tests

Pulmonary function measures were obtained using a pneumotachograph with a volume integrator (CAD/Net system 1070; Medical Graphics Corporation, St. Paul, Minn., United statesSA), following American Thoracic Society criteria (Clausen et al., 1997). Spirometric parameters were expressed as percent of predicted values and z-scores. Predicted values and z-scores were derived using prediction equations from the Global Lung Function Initiative (GLI-2012; http://www.lungfunction.org/) (Quanjer et al., 1993; Quanjer et al., 2012). Only pre-bronchodilator data were included in the study data analysis.

### Bronchial Provocation Test

A methacholine challenge test was performed as a dose-response curve by increasing (doubling) doses of methacholine chlorohydrate every 3 min according to international guidelines (Pizzichini et al., 1999). Results were expressed as cumulative doses of methacholine provoking a 20% fall in FEV_1_ (PD_20_ FEV_1_). A methacholine challenge test result was considered positive if the PD_20_ FEV_1_ was <16.00 mg/ml (Crapo et al., 2000).

### F_E_NO

F_E_NO was determined with a high-resolution chemiluminescence NO analyser (Ecomedics AG Analyzer CLD88; Dürnten, Switzerland), with detection limit of 0.06 ppb and measurement range reaching 100 ppb. FENO was measured at a flow rate of 50 ml/s as per ATS/ERS guidelines. Measurements were obtained in accordance with the ATS recommendations for on-line measurement of F_E_NO in adults (American Thoracic Society, European Respiratory Society, 2005).

### Sputum Induction

After baseline FEV_1_ and FVC measurements, subjects were pre-treated with inhaled salbutamol (200 μg by metered-dose inhaler) and 10 min later were asked to inhale a hypertonic (4.5%) nebulized sterile saline solution for three periods of 5 min each at most by means of an ultrasonic nebulizer (Ultraneb 2000; DeVilbiss, Somerset, PA, USA). Nebulization was discontinued if one of the following symptoms occurred: wheezing, chest tightness or moderate-to-severe dyspnea. Sputum was processed as previously reported (Malerba et al., 2006). The cut-off for an abnormal result was considered a sEOS% value > 3% of total non-squamous cells (Balbi et al., 2007).

### Statistical Analysis

In an opportunistic sample of 310 young adults with CVA, we aimed to provide a proof of concept for the use of F_E_NO, sEOS%, FEF_25–75%_ predicted value, and FEF_25–75%_ z scores as surrogate markers predicting BHR. Subject characteristics have been summarized according to BHR status. The normality Shapiro Wilk test was performed for assessing data distribution. Normally distributed data were expressed as mean and the standard deviation (SD); nonparametric data were expressed as median and interquartile range (25^th^ to 75^th^ percentiles); categorical data were expressed as percentage and absolute numbers. Wilcoxon rank sum test or *t*-test, depending on data distribution, was performed for continuous data between-group comparisons; Pearson chi-square test or Fisher-exact test, whatever appropriate, was used for categorical variable between-group comparisons. Correlations between PD_20_ FEV_1_, sEOS% and F_E_NO were expressed as Spearman Rho correlation coefficient. FEF_25–75%_ was also expressed as a z-score using the regression equation and variance derived from a normal population assessed in our laboratory. FEF_25–75%_ z-score was calculated as the difference between the measured and predicted FEF_25–75%_ value divided by the reference SD (Jones et al., 2003). A Z score that equaled zero indicated the subject's pulmonary function was at the predicted value, whereas Z scores of 1 and −1 indicated pulmonary function that was 1 SD above and below the predicted values, respectively.

The predictive accuracies for each BHR predictor have been estimated via logistic regression models. The estimates have been adjusted by gender and age and validated performing a 10 fold repeated cross-validation procedure. Receiver operating characteristic (ROC) curves, sensitivity and specificity values with relative standard deviations computed across iterations have been reported. ROC curves for the leading predictors have also been reported.

Optimal cut off values (OCV) for BHR prediction were estimated as the values combining the best sensitivity and specificity for BHR POS detection. The Youden index (J), a main summary statistic of the ROC curve, was used as a measure of model quality (Youden, 1950). Statistical analysis has been performed using R 3.2.5 (R Core Team., 2018), together with caret (Kuhn, 2008) and pROC packages (Robin et al., 2011).

## Results

### Baseline Characteristics of Subjects Studied

Clinical data from 310 subjects were included in the analysis. Subjects were divided in two groups based on methacholine BPT results. Subjects with positive BPT were categorized as BHR POS (*n* = 147); if BPT was negative, subjects were identified as BHR NEG (n = 163).

There was no between-group difference in age (*p* = 0.36) and gender (*p* = 0.73), whereas allergy was more prevalent in BHR POS (68%) than in BHR NEG (6%) (*p* < 0.001) (Table 1). One hundred ten subjects (35%) were sensitized to perennial and/or pollen allergens. Subjects were all ex smokers or non-smokers.

**TABLE 1:** Subject characteristics in positive and negative bronchial hyperreactivity (BHR) groups.

	BHRneg	BHRpos	Combined	*p*
Number	163	147	310	
Female	64% (104)	62% (91)	63% (195)	0.73
Age, years	25.1 (31.1–35.8)	23.3 (29.4–35.2)	24.0 (30.0–35.7)	0.36
Allergy	6% (10)	68% (100)	35% (110)	<0.001
ICS treatment, yes/no	0/163	0/147	0/310	
History of smoking, yes/no	0/163	0/147	0/310	
FEV1, % pred	97.8 (98.7–99.4)	98.0 (99.1–100.0)	97.9 (98.9–99.7)	0.064
FVC, % pred	98.3 (99.2–100.3)	98.4 (99.5–101.1)	98.3 (99.3–100.7)	0.059
FEV1/FVC, %	0.820 (0.830–0.840)	0.820 (0.830–0.850)	0.820 (0.830–0.850)	0.76
FEF_25–75%_, z-score	−0.605 (0.37–0.76)	−2.77 (−1.96–−1.14)	−2.005 (−0.815–0.445)	<0.001
FEF25–75%, % pred	86 (110–120)	44 (57–72)	56 (81–111)	<0.001
FENO, ppb	17.8 (19.3–21.9)	42.5 (56.6–63.0)	19.0 (28.0–55.0)	<0.001
sEOS, %	0.0 (0.0–1.4)	6.4 (7.9–11.7)	0.0 (2.6–7.6)	<0.001
PD20 FEV1	1600/1600/1600	195/570/1000	605/1600/1600	<0.001

Continuous data are reported as median and interquartile range (25^th^ to 75^th^ percentiles); categorical data are reported as a percentage and numbers. Wilcoxon rank sum test was performed for continuous variable comparisons and the Pearson chi-square test or Fisher-exact test, whatever appropriate, was used for categorical variable comparisons. Abbreviations: BHR, bronchial hyperresponsiveness; ICS, inhaled corticosteroids; FEV1, forced expiratory volume in 1 s; FVC, forced vital capacity; FEF25–75%, forced expiratory flow between 25 and 75% of FVC; FENO, fractional exhaled nitric oxide; pred: predicted value; sEOS%, percentage of eosinophil differential cell count in induced sputum.

### Functional and Inflammatory Biomarkers

In all subjects, FVC, FEV_1_ and FEV_1_/FVC values were within normal reference ranges showing no significant differences between BHR POS and BHR NEG group (Table 1).

By contrast, median FEF_25–75%_ z-score (*p* < 0.001) and FEF_25–75%_ % predicted value (*p* < 0.001) were both significantly lower in the BHR POS than BHR NEG group (Table 1), suggesting that small airways disease was present in the former.

F_E_NO (*p* < 0.001) and sEOS% median values (*p* < 0.001) were elevated in the BHR POS group compared with BHR NEG group (Table 1). Mean PD_20_ FEV_1_ was 642 ± 489 μg. F_E_NO concentrations were correlated with PD_20_ FEV_1_ values (rho = −0.88; *p* < 0.001). F_E_NO and sEOS% values were highly correlated (rho = 0.886 *p* < 0.001).

### Diagnostic Accuracy of Single Measurements for BHR Prediction

**FIGURE 1:**
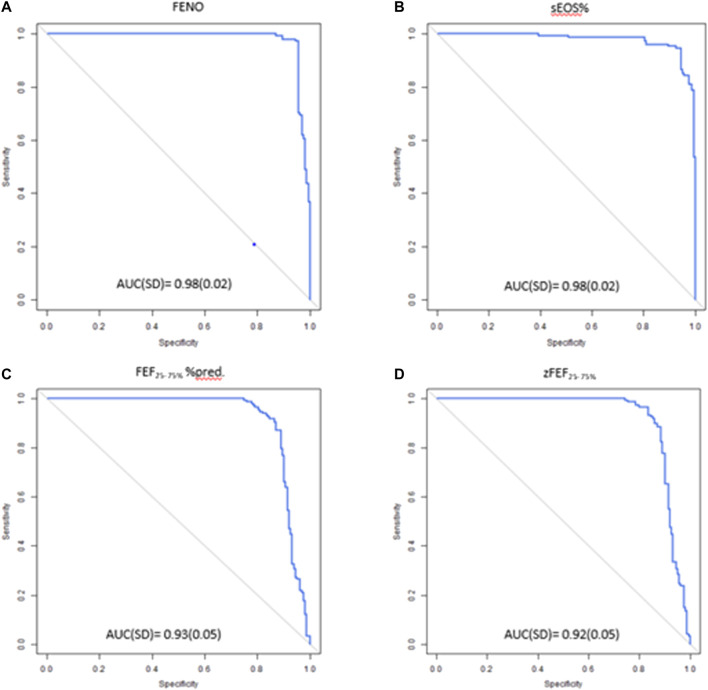
ROC curves of **(A)** F_E_NO **(B)** sEOS% **(C)** FEF_25–75%_%pred **(D)** zFEF_25–75%_ in predicting positive BHR. ROC, receiver operating characteristic; F_E_NO, fractional exhaled nitric oxide; sEOS%, percentage of eosinophils in induced sputum; FEF_25–75%_, forced expiratory flow at 25–75% of forced vital capacity: AUC, Area under the curve; SD standard deviation.

Logistic regression analysis showed that measurement of F_E_NO, sEOS%, FEF_25–75%_ z-score, and FEF_25–75%_ % predicted values were able to predict BHR with high accuracy, sensitivity and specificity (Table 2). F_E_NO and sEOS% were the best performing techniques as reflected by their AUC values (0.98 for both) (Figure 1), and sensitivity and specificity values, which were above 0.92 (Table 2). AUCs for FEF_25–75%_ % pred and FEF_25–75%_ z-score were >0.91, with sensitivity and specificity values > 0.86 (Figure 1; Table 2).

**TABLE 2:** BHR predictive accuracies of F_E_NO measurement, percentage of sputum eosinophil cell counts, FEF_25%–75%_ of predicted value, and FEF_25%–75%_ z-score in 310 subjects with suspected cough variant asthma.^a^

	AUC	AUC SD	Sens	Sens SD	Spec	Spec SD	Cut off	PPV	NPV	Accuracy	PLR	NLR	Youden index
F_E_NO	0.98	0.02	0.93	0.07	0.96	0.05	32.70	0.95	0.93	0.94	21.56	0.08	0.89
sEOS	0.98	0.02	0.94	0.07	0.94	0.05	3.80	0.94	0.95	0.94	17.01	0.06	0.88
FEF25–75% % pred	0.93	0.05	0.90	0.07	0.87	0.07	80.00	0.86	0.91	0.89	6.96	0.11	0.77
FEF25–75% z-score	0.92	0.05	0.89	0.09	0.87	0.09	−0.87	0.86	0.90	0.88	6.71	0.12	0.76

^a^ Area under the receiver operator characteristic (ROC) curve (AUC), sensitivity, specificity, with standard deviations (SD), optimal cut-points (cutoff), negative and positive predictive values, and likelihood ratios are shown. The predictive accuracies have been estimated via logistic regression models adjusted by gender and age, performing a 10 fold repeated (10 times) cross-validation procedure. Abbreviations: AUC, area under receiver operator characteristic (ROC) curve; SD, standard deviation; Sens, sensitivity; spec, specificity; PPV, positive predictive value; NPV, negative predictive value; PLR, positive likelihood ratio; NRL, negative likelihood ratio; FENO: fractional exhaled nitric oxide; sEOS, eosinophil count in induced sputum; FEF25%-75%, forced expiratory flow between 25 and 75% of forced vital capacity; pred, predicted.

OCV for BHR prediction were as follows: F_E_NO, 32.7 ppb (sensitivity = 0.93, specificity = 0.96), sEOS%, 3.80% (sensitivity = 0.94, specificity = 0.94), FEF_25–75%_ % predicted, 80.0% (sensitivity = 0.90, specificity = 0.87), and FEF_25–75%_ z-score, -0.87 (sensitivity = 0.89, specificity = 0.87). Youden J values of various techniques for predicting BHR are shown in Table 1.

## Discussion

Our study provided proof of concept that non-invasive/semi-invasive measures reflecting airway inflammation or small-airway function might be used as surrogate markers of BHR in young adults with suspected CVA and normal lung function. Predictive models were of high quality as reflected by values of Youden’s index (J), the maximum potential effectiveness of a biomarker which combines sensitivity and specificity (Youden, 1950), ranging from 0.76 to 0.89. We confirmed the potential utility of F_E_NO and FEF_25–75%_ predicted value measurement in predicting the presence of BHR in subjects with suspected CVA and extend this observation to eosinophil counts in induced sputum. Of note, in our study, the discriminant abilities of the various methods were remarkably higher than those reported in previous studies (Schleich et al., 2012; Bao et al., 2018; Chen et al., 2019), This discrepancy might reflect differences in study population characteristics, including age, ethnicity, and lung function, across various studies.

We observed that young adults with suspected CVA and normal lung function having F_E_NO >37.2 ppb, sEOS% >3.8%, FEF_25–75%_ % pred <80% and zFEF_25–75%_ <-0.87% have elevated probability of being BHR POS and classification accuracy >91% (98% for both F_E_NO and sEOS%). These findings support a close relationship between airway inflammation and peripheral airway function.

F_E_NO is a surrogate marker of airway inflammation, particularly useful in patients with atopic eosinophilic asthma (Berkman et al., 2005; Gibson, 2009; Dweik et al., 2011). FeNO was proposed to be able also to predict ICS responsiveness in chronic cough although supported by few studies and without a strong evidence (Song et al., 2017b).

In individuals with chronic cough (Maniscalco et al., 2015; Chen et al., 2019), F_E_NO is able to distinguish CVA and eosinophilis bronchitis from other causes of chronic cough. A recent meta-analysis pointed out that Fractional exhaled nitric oxide (FeNO) has moderate diagnostic accuracy for predicting cough variant asthma (CVA) with high specificity in patients with chronic cough (Song et al., 2017a). We observed a strong correlation between F_E_NO and sEOS% values consistent with previous reports on the ability of F_E_NO to reflect eosinophilic airway inflammation (Cirillo et al., 2013; Ricciardolo, 2014). In the present study, F_E_NO showed a remarkable predictive ability to detect BHR (AUC: 0.98; sensitivity: 0.93; specificity: 0.96; PPV: 0.95; NPV: 0.93) at a cutoff value of >32.7 ppb, higher than that reported in previous studies at F_E_NO cutoff values of 43 ppb (AUC: 0.79; sensitivity: 0.72; specificity: 0.82; PPV: 0.66; NPV: 0.5) (Bao et al., 2018), 34 ppb (AUC: 0.62; PPV: 0.88; NPV: 0.62) (Schleich et al., 2012), and 25 ppb (AUC: 0.65; PPV: 0.83; NPV: 0.49) (Bougard et al., 2020), and others (Song et al., 2017a; Chen et al., 2019). These discrepancies might be explained, at least partly, by population study differences, including ethnicity (all Caucasian population vs. all Chinese population) (Bao et al., 2018), mean age (24 years vs. 43 (Bao et al., 2018) or 41 (Schleich et al., 2012) or 51 (Bougard et al., 2020) years), smoking habit (nonsmokers vs. 6–19% (Bao et al., 2018) or 34% (Schleich et al., 2012) current smokers), atopy (35 vs. 93% (Schleich et al., 2012), not reported in reference 6), symptoms (suspected CVA vs. suspected asthma with negative bronchodilator reversibility test (Schleich et al., 2012), and inhaled corticosteroid (ICS) treatment (Bougard et al., 2020). Atopy seems to paly a robust role as in a recent paper the diagnostic accuracy of F_E_NO for predicting CVA in chronic cough in patients with atopy was clearly higher than in patients without (Chen et al., 2019). Based on our data, F_E_NO might have a greater predictive value in distinguishing subjects with or without BHR in young adults with undifferentiated cough and normal lung function. In this population, the high BHR predictive accuracy of F_E_NO measurement (AUC: 0.98; PPV: 0.95; NPP: 0.93; PLR: 21.56; NLR: 0.08) suggest that BPT should be limited to individuals with F_E_NO > 32.7 ppb. In this perspective F_E_NO could be used as a rule-in test for CVA as previously suggested (Song et al., 2017a).

Measurement of percentage of eosinophil cell counts in induced sputum as a candidate for predicting BHR is novel. In the present study, at a cutoff value of 3.8%, this method showed higher accuracy and BHR predicting capacity (AUC: 0.98; PPV: 0.94; NPV: 0.95) than peripheral blood eosinophil cell counts at a cutoff value of 3.5% (AUC: 0.76; PPV: 58.9; NPV: 85.1) (Bao et al., 2018). Apart from variations in study populations, this inconsistency might derive from methodological differences, as measurement of eosinophil cell counts in induced sputum is a direct and likely more accurate measure of airway inflammation than peripheral blood eosinophils. Along with F_E_NO, sEOS% showed the highest discriminative capacity to identify BHR POS individuals under our experimental conditions. However, measurement of sEOS% is not available in all centers as it requires trained and experienced staff for sputum induction, processing and analysis.

Classification above a threshold value of 70% is considered significant (Bijlsma et al., 2006). In the present study, FEF_25–75%_ measures showed significant discriminative capabilities as reflected by AUCs >0.91 with PPV >0.85 and NPV >0.89 at OCV. FEF_25–75%_ has been found to correlate with functional imaging assessment of small airway function (Jain et al., 2005). Small airways disease plays a relevant role in asthma pathophysiology (Van Der Wiel et al., 2013; van der Wiel et al., 2014). Airway wall thickening induced by inflammation, airway narrowing, and enhanced airway muscular tone contribute to small airway dysfunctions and poorly controlled asthma (van der Palen et al., 2013). Measurement of FEF_25–75%_ to detect small airway dysfunction in asthmatic subjects with normal FEV_1_ values could be a useful diagnostic tool (Malerba et al., 2016). Our findings, showing that the FEF_25–75%_ % predicted values in BHR POS individuals are lower than those observed in BHR NEG individuals, confirm the presence of small airway disease in subjects with FVC, FEV_1_, and FEV_1_/FVC values within normal limits. Compared with F_E_NO at cutoff of 32.7 ppb (AUC: 0.98; sensitivity: 0.93), FEF_25–75%_ % predicted value showed a lower discriminative capacity at a cutoff of 80% (AUC: 0.93), but a similar sensitivity (0.90) using 80% as cutoff value. If inflammatory measures such as F_E_NO and sEOS% are not available, a FEF_25–75%_ > 80% predicted value could help rule out CVA diagnosis in young adults with cough and aid clinicians in diagnosing BHR positive subjects referring to BPT only subjects with FEF_25–75%_ < 80% predicted values.

Strengths of our study are represented by assessment and comparison of both small airway function and inflammatory potential surrogate markers of BHR, including measurement of eosinophil counts in induced sputum, and inclusion of a large cohort of young adults with suspected CVA and normal lung function as reflected by FEV_1_ values.

Inclusion of a relatively homogeneous study population consisting of non-smoker, steroid-naïve, individuals with a median age of 24 years represents a strength, but, at the same time, a study limitation as it precludes the assessment of the impact of confounding factors, including age, ethnicity, smoking habit, comorbidities, and ICS treatment on study outcomes. These findings cannot be generalized. F_E_NO concentration reliably reflects central airway inflammation, but is not generally considered to reflect peripheral airway inflammation for which estimating alveolar NO concentration (CANO) by measurement of F_E_NO at different flow rates could be more useful (Lehtimäki et al., 2020). Other study limitations include the lack of internal validation of results with training and testing datasets and external validation, the higher prevalence of atopy in BHR POS than in BHR NEG subjects (68 vs. 6%, respectively), which may have influenced the outcomes of F_E_NO and sEOS% diagnostic performance, the lack of assessment of air pollution exposure potential impact and objective measures of smoking status, and the absence of post-bronchodilator data. However, the latter is not usually required in individuals with FEV_1_ >80% predicted value. As our study was conducted in young adults, results and their interpretation should be limited to this age group. Further research is warranted to determine if these results can be generalized to older individuals with asthma.

Finally, assessment of small airway function was limited to FEF_25–75%_. However, other functional measure, including nitrogen washout and plethysmography, are not routinely used in clinical practice.

In conclusion, our study shows elevated capacities of noninvasive/semi-invasive methods, particularly F_E_NO and sEOS%, in predicting BHR in young adults with suspected CVA and normal lung function, and points out the importance of the target population choice in determining their diagnostic performances. External validation of these research outcomes in independent cohorts is required before translating this approach into clinical decision-making.

## Data Availability

The datasets presented in this article are not readily available because available upon reasonable request; Requests to access the datasets should be directed to mario.malerba@uniupo.it.
